# VISTA expression is associated with a favorable prognosis in patients with high-grade serous ovarian cancer

**DOI:** 10.1007/s00262-019-02434-5

**Published:** 2019-11-28

**Authors:** Liju Zong, Yuncan Zhou, Ming Zhang, Jie Chen, Yang Xiang

**Affiliations:** 1 Department of Obstetrics and Gynecology, Peking Union Medical College Hospital, Chinese Academy of Medical Sciences and Peking Union Medical College, No.1 Shuaifuyuan, Dongcheng District, Beijing, 100730 China; 2 Department of Pathology, Peking Union Medical College Hospital, Chinese Academy of Medical Sciences and Peking Union Medical College, Beijing, China

**Keywords:** Ovarian cancer, PD-L1, VISTA, Immune checkpoints, Prognosis

## Abstract

Blockading programmed death ligand 1 (PD-L1) shows promising results in patients with some cancers, but not in those with ovarian cancer. V-domain Ig suppressor of T cell activation (VISTA) is a recently discovered immune checkpoint protein that suppresses T cell activation. This study aimed to investigate the expression and clinical significance of VISTA in ovarian cancer as well as its relationship with PD-L1. VISTA and PD-L1 levels in 146 ovarian cancer samples were assessed using immunohistochemistry. We investigated the association between VISTA and other clinicopathological variables, including survival. The associations between the VISTA-encoding *C10orf54* gene, other immune checkpoints, and survival were analyzed. VISTA was detected in 51.4% of all samples and 46.6% of PD-L1-negative samples; it was expressed in 28.8%, 35.6%, and 4.1% of tumor cells (TCs), immune cells (ICs), and endothelial cells, respectively. Furthermore, VISTA expression was associated with pathologic type and PD-L1 expression. Moreover, VISTA expression in TCs, but not in ICs, was associated with prolonged progression-free and overall survival in patients with high-grade serous ovarian cancer. The expression of *C10orf54* mRNA was associated with prolonged overall survival and immune escape-modulating genes. These results showed that VISTA expression in ovarian tumor cells was associated with a favorable prognosis in patients with high-grade serous ovarian cancer; however, additional studies are required to better understand the expression and role of VISTA in ovarian cancer.

**Electronic supplementary material:**

The online version of this article (10.1007/s00262-019-02434-5) contains supplementary material, which is available to authorized users.

## Introduction

Ovarian cancer is the most lethal type of the gynecologic malignancy and accounts for the highest rate of gynecological cancer-related deaths worldwide. Approximately 295,414 patients were newly diagnosed with ovarian cancer in 2018, and 184,799 women died of this disease [1]. More than 75% of patients are diagnosed at an advanced stage (stage III/IV), for which the 5-year overall survival (OS) rate is less than 30%. Although surgery and adjuvant chemotherapy are effective for the majority of patients with ovarian cancer, more than 70% ultimately relapse, and their tumors eventually become chemotherapy resistant.

Immunotherapy with antibodies against negative immune checkpoints such as programmed death 1 (PD-1), its ligand PD-L1, and cytotoxic T-lymphocyte-associated protein 4 (CTLA4) has shown great efficacy in patients with a variety of solid tumors [2]. However, in a study of 26 patients with PD-L1-expressing advanced ovarian cancer, the objective response rate to immunotherapy was 11.5% (only one patient achieved a complete response and two had partial responses) [3]. Therefore, there is an urgent need to improve the outcomes of patients with ovarian cancer by better understanding the immune microenvironment of this tumor type and developing effective immunotherapeutic strategies [4].

V-domain Ig suppressor of T cell activation (VISTA) is a protein encoded by the *C10orf54* gene and is also known as PD-1 homolog. VISTA is a recently discovered inhibitory immune checkpoint molecule that belongs to the B7 family and shares sequence homology with both PD-1 and PD-L1 [5–7]. This protein can suppress T cell activation when expressed as a ligand on antigen-presenting cells or when expressed as a receptor on T cells [8, 9]. Liu and colleagues reported a nonredundant, T cell-activating role for VISTA that is distinct from the PD-1/PD-L1 pathway [8]; this provided a rationale for the combined targeting of the VISTA and PD-1 pathways when treating patients with cancer.

Interestingly, Kakavand et al. discovered a significantly higher density of VISTA-positive lymphocytes after treatment with an anti-PD-1 antibody alone or in combination with anti-CTLA4 (ipilimumab); this was associated with adaptive resistance to immune checkpoint blockade [10]. Similarly, Gao et al. found that VISTA expression was elevated after anti-CTLA4 therapy in patients with prostate cancer, suggesting that elevated VISTA expression is a compensatory event in the setting of ipilimumab therapy [11]. When comparing immune cell (IC) infiltrates in melanoma and pancreatic cancer, Blando et al. demonstrated that VISTA is preferentially expressed at higher levels in pancreatic cancer, and highlighted this protein as a potential immunotherapeutic target for patients with this disease [12]. Furthermore, preclinical murine studies have shown that blockading both VISTA and PD-L1 produces a synergistic therapeutic effect in colon cancer models [8]. These studies suggest that VISTA may modulate a novel immune evasion mechanism and is thus a potential target for cancer immunotherapy. VISTA expression in human cancers has been reported in non-small cell lung cancer (NSCLC), hepatocellular carcinoma, colorectal carcinoma, oral squamous cell carcinoma, gastric carcinoma, acute myeloid leukemia, and gestational trophoblastic neoplasia [13–21]. Mulati et al. also found that VISTA was highly expressed in human ovarian and endometrial cancers [20]. However, the relationships between VISTA, PD-L1, clinicopathologic features, and prognoses in patients with ovarian cancer remain unknown.

In the current study, we investigated VISTA and PD-L1 expression using a tumor tissue microarray (TMA) encompassing the major histological subtypes of ovarian cancer. To support our findings, we also performed analyses of The Cancer Genome Atlas (TCGA) ovarian cancer dataset.

## Materials and methods

### Patient cohort and TMA

The commercialized TMA (panel HOvaC160Su01) and clinical data were provided by Shanghai Outdo Biotech Co. Ltd. (Shanghai, China). The TMA comprised samples from 160 patients with stage I–IV ovarian cancer that were collected between 2009 and 2012. All tumor tissues were obtained at the time of primary surgery from patients who were deemed optimally debulked. A total of 146 samples were included in this analysis after excluding non-epithelial ovarian cancer samples and quality controls. Of these samples, 93 were high-grade serous ovarian carcinoma (HGSOC), 27 were mucinous ovarian carcinoma, 17 were endometrioid ovarian carcinoma, and 9 were ovarian clear cell carcinoma. When categorized by International Federation of Gynecology and Obstetrics (FIGO) stage, eight samples were stage I, 31 were stage II, 75 were stage III, and 32 were stage IV. The mean age of the patients was 51 years (range 23–75 years), and the median follow-up time was 41 months (range 4–109 months).

### Immunohistochemistry

Primary antibodies against VISTA (clone D1L2G, 1:200; Cell-Signaling Technology, MA, USA) and PD-L1 (clone E1L3 N, 1:200; Cell-Signaling Technology, MA, USA) were used to detect the expression of VISTA and PD-L1. Human tonsil and placenta tissues that were obtained from the Department of Pathology, Peking Union Medical College Hospital, Beijing, China, were used as positive controls, whereas normal tonsil tissues without primary antibodies were used as negative controls. Immunohistochemistry was performed using our laboratory protocol as described previously [13, 22].

Samples stained for VISTA and PD-L1 were evaluated by two pathologists who were blinded to the clinical outcomes. PD-L1 expression was evaluated with binary positive/negative scoring; PD-L1 positivity was defined as membranous staining on ≥ 1% of the cells using the previously described ‘combined positive score’ [23], which is calculated by summing the number of PD-L1-stained cells (tumor cells [TCs], lymphocytes, and macrophages), dividing the result by the total number of viable TCs, and multiplying the quotient by 100 [23]. VISTA expression was evaluated in both TCs and tumor-infiltrating ICs; TCs were considered VISTA-positive if at least 1% of these cells per histospot had membranous and/or cytoplasmic staining. ICs were defined as VISTA-positive if any staining was present in at least 1% of these cells, which included macrophages and lymphocytes. Endothelial cells were designated positive if any VISTA staining was present within. For purposes of stratification and statistical analysis, VISTA in each sample was defined as positive if any staining was visible in the TCs, ICs, or endothelial cells.

### Kaplan–Meier plotter analysis

To analyze the prognostic value of mRNA from the VISTA-encoding gene *C10orf54* in HGSOC, we performed survival analysis using the Kaplan–Meier plotter (www.kmplot.com), which contains gene expression data and patient survival information related to 2190 ovarian cancers derived from the Gene Expression Omnibus, European Genome–Phenome Archive, and TCGA. Patients with HGSOC (grades 2–3) who had stage II–IV disease were included in this study for the analysis of OS (*n* = 405) and progression-free survival (PFS) (*n* = 402). Patient samples were split into two groups (high vs. low expression) according to the most optimal cutoff of *C10orf54* mRNA levels, which was automatically determined by the Kaplan–Meier plotter. The survival rates of patients in the two groups were compared using Kaplan–Meier plots; the hazard ratios, 95% confidence intervals, and log-rank *P* values were calculated [24].

### TCGA data analysis for mRNA expression

We analyzed VISTA mRNA expression in the ovarian serous cystadenocarcinoma samples from the TCGA database using cBioPortal for cancer genomics (http://www.cbioportal.org/) [25, 26]. We included a total of 291 samples with the next-generation sequencing data (RNA Seq V2 RSEM), and evaluated the association between log2-transformed mRNA ‘fragments per kilobase million’ scores of the genes encoding VISTA, CD8A, and other inhibitory immune checkpoints including CTLA4, PD-1, PD-L1, PD-L2, T cell immunoglobulin and mucin domain-3 (TIM-3), lymphocyte activation gene-3 (LAG3), and T cell immunoreceptor with Ig and ITIM domains (TIGIT) according to the cBioPortal instructions [26]. In addition, co-expressed genes with Pearson correlation coefficients > 0.4 were used for gene-ontology enrichment annotation using the cluster Profiler package [27].

### Statistical analysis

The Chi-square or Fisher’s exact test was used to analyze the correlations between categorical variables (VISTA, PD-L1, and pathologic subtypes). Survival curves were plotted using the Kaplan–Meier method and compared using the log-rank test. Statistical analyses were performed using SPSS 20.0 for Windows (IBM Corp., Armonk, NY, USA). Graphs and images were prepared using Prism 5.0 (GraphPad Software, La Jolla, CA, USA) and Photoshop CC 2019 for Windows (Adobe Inc., San Jose, CA, USA). All statistical tests were two-sided, and *P* values < 0.05 were considered significant.

## Results

### VISTA and PD-L1 expression in epithelial ovarian cancer

**Fig. 1:**
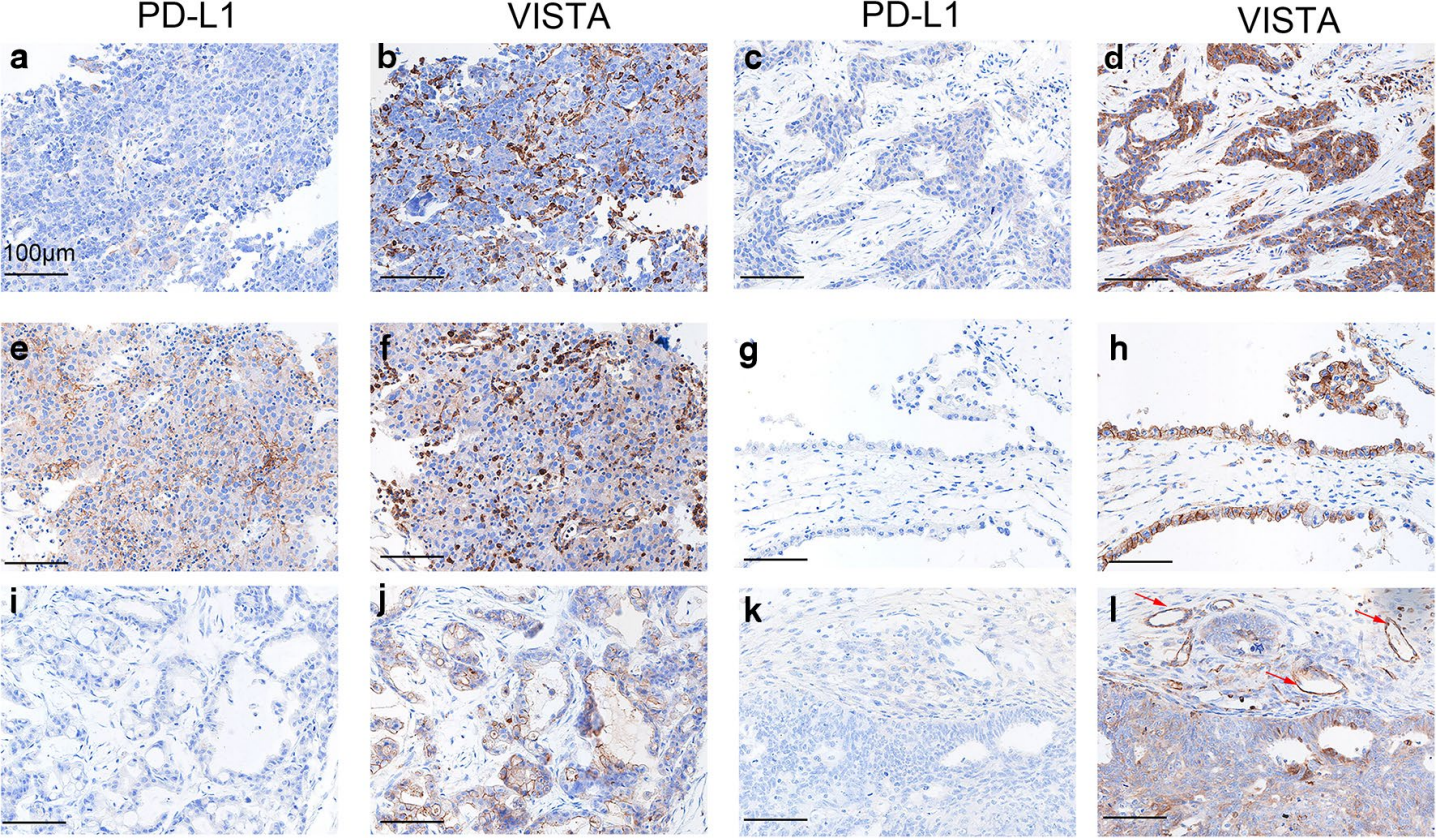
Representative immunohistochemical staining of V-domain Ig suppressor of T cell activation (VISTA) and PD-L1 in human ovarian cancer. **a**, **b** Negative PD-L1 and positive VISTA in immune cells (ICs) in high-grade serous ovarian carcinomas (HGSOC). **c**, **d** Negative PD-L1 and positive VISTA in tumor cells (TCs) and ICs in HGSOC. **e**, **f** Positive PD-L1 and positive VISTA in ICs in HGSOC. **g**, **h** Negative PD-L1 and positive VISTA in TCs in clear cell carcinoma. **i**, **j** Negative PD-L1 and positive VISTA in TCs in mucinous ovarian carcinoma. **k**, **l** Negative PD-L1 and positive VISTA in TCs, ICs, and endothelial cells (red arrows) in endometrioid carcinoma

Among the 146 samples overall, VISTA expression was detected in 51.4% (75 samples), while PD-L1 was expressed in 8.9% (13 samples). VISTA protein was detected in TCs, ICs, and endothelial cells, and exhibited a cytoplasmic/membranous staining pattern. Representative stained samples of PD-L1 and VISTA are shown in Fig. 1. Forty-two samples (28.8%) showed VISTA expression in TCs, 52 (35.6%) showed its expression in ICs, and 6 (4.1%) had VISTA-positive endothelial cells. VISTA expression in the four types of ovarian cancer is shown in Table 1. Notably, 46.6% (62/133) of the PD-L1-negative samples expressed VISTA. When comparing non-serous carcinoma with HGSOC, VISTA expression levels in both the TCs and ICs of mucinous carcinomas were lower than those in the TCs and ICs of serous carcinomas; moreover, VISTA expression in the ICs of clear cell carcinomas was lower than that in the ICs of serous carcinomas (Table 1). However, no differences in VISTA expression were observed between serous carcinoma and endometrioid ovarian carcinoma.

**Table 1:** VISTA expression in the four pathologic types of ovarian cancer

VISTA expression	Serous carcinoma	Mucinous carcinoma	Endometrioid carcinoma	Clear cell carcinoma
	*n* (%)	*n* (%)	*n* (%)	*n* (%)
VISTA in all cells	/	P < 0.001	*P* = 0.272	*P* = 0.751
Positive	57 (61.3)	4 (14.8)	8 (47.1)	6 (66.7)
Negative	36 (38.7)	23 (85.2)	9 (52.9)	3 (33.3)
VISTA in ICs	/	P < 0.001	*P* = 0.149	P = 0.004
Positive	45 (48.2)	2 (7.4)	5 (29.4)	0 (0)
Negative	48 (51.6)	25 (92.6)	12 (70.6)	9 (100)
VISTA in TCs	/	P = 0.016	*P* = 0.670	*P* = 0.057
Positive	28 (30.1)	2 (7.4)	6 (35.3)	6 (66.7)
Negative	65 (69.9)	25 (92.6)	11 (64.7)	3 (33.3)

*ICs* immune cells, *TCs* tumor cells, *VISTA* V-domain Ig suppressor of T cell activation

### VISTA expression is associated with favorable prognoses in patients with HGSOC

We investigated the associations between VISTA expression, FIGO stage, PD-L1 expression, and survival in patients with HGSOC. The expression of VISTA was positively correlated with that of PD-L1. VISTA expression in ICs as well as in all cells (TCs, ICs, and endothelial cells) combined was significantly more frequent in PD-L1-positive cells. However, VISTA expression in TCs alone was not associated with PD-L1 expression (Table 2).

**Table 2:** Association between VISTA expression, stage, PD-L1, and survival in patients with high-grade serous ovarian cancer

		VISTA in all cells		VISTA in ICs		VISTA in TCs	
	Total	Positive	Negative	Positive	Negative	Positive	Negative
	*n* (%)	*n* (%)	i (%)	*n* (%)	*n* (%)	*n* (%)	*n* (%)
Stage	93	*P* = 0.289		*P* = 0.794		*P* = 0.146	
I	3 (3.2)	1	2	1	2	0	3
		(33.3)	(66.7)	(33.3)	(66.7)	0	(100)
II	18 (19.4)	14	4	10	8	8	10
		(77.8)	(22.2)	(55.6)	(44.4)	(44.4)	(55.6)
III	44 (47.3)	27	17	22	22	15	29
		(61.4)	(38.6)	(50)	(50)	(34.1)	(65.9)
IV	28 (30.1)	15	13	12	16	5	23
		(53.6)	(46.4)	(42.9)	(57.1)	(17.9)	(82.1)
PD-L1		P = 0.006		P = 0.018		*P* = 0.297	
Positive	11 (11.8)	11	0	9	2	5	6
		(100)	(0)	(81.8)	(18.2)	(45.5)	(54.5)
Negative	82 (88.2)	46	36	36	46	23	59
		(56.1)	(43.9)	(43.9)	(56.1)	(28)	(72)
PFS		*P* = 0.151		*P* = 0.201		P = 0.043	
Mean (months)	38.4	42	32.8	42.9	34.5	46.4	33.8
95% CI	31.6–45.2	33.1–50.9	22.8–42.9	32.7–53.2	25.6–43.3	35.0–57.8	26.2–41.3
OS		*P* = 0.225		*P* = 0.427		P = 0.040	
Mean (months)	57.2	60.8	51.2	60.3	54.5	71.1	51.2
95% CI	49.2–65.2	50.8–70.9	43.1–68.1	38.5–64.0	43.8–65.3	58.5–83.6	41.5–60.9

“All cells” include ICs, TCs, and endothelial cells

*PD-L1* programmed cell death ligand 1, *PFS* progression-free survival, *CI* confidence interval, *OS* overall survival, *ICs* immune cells, *TCs* tumor cells, *VISTA* V-domain Ig suppressor of T cell activation

**Fig. 2:**
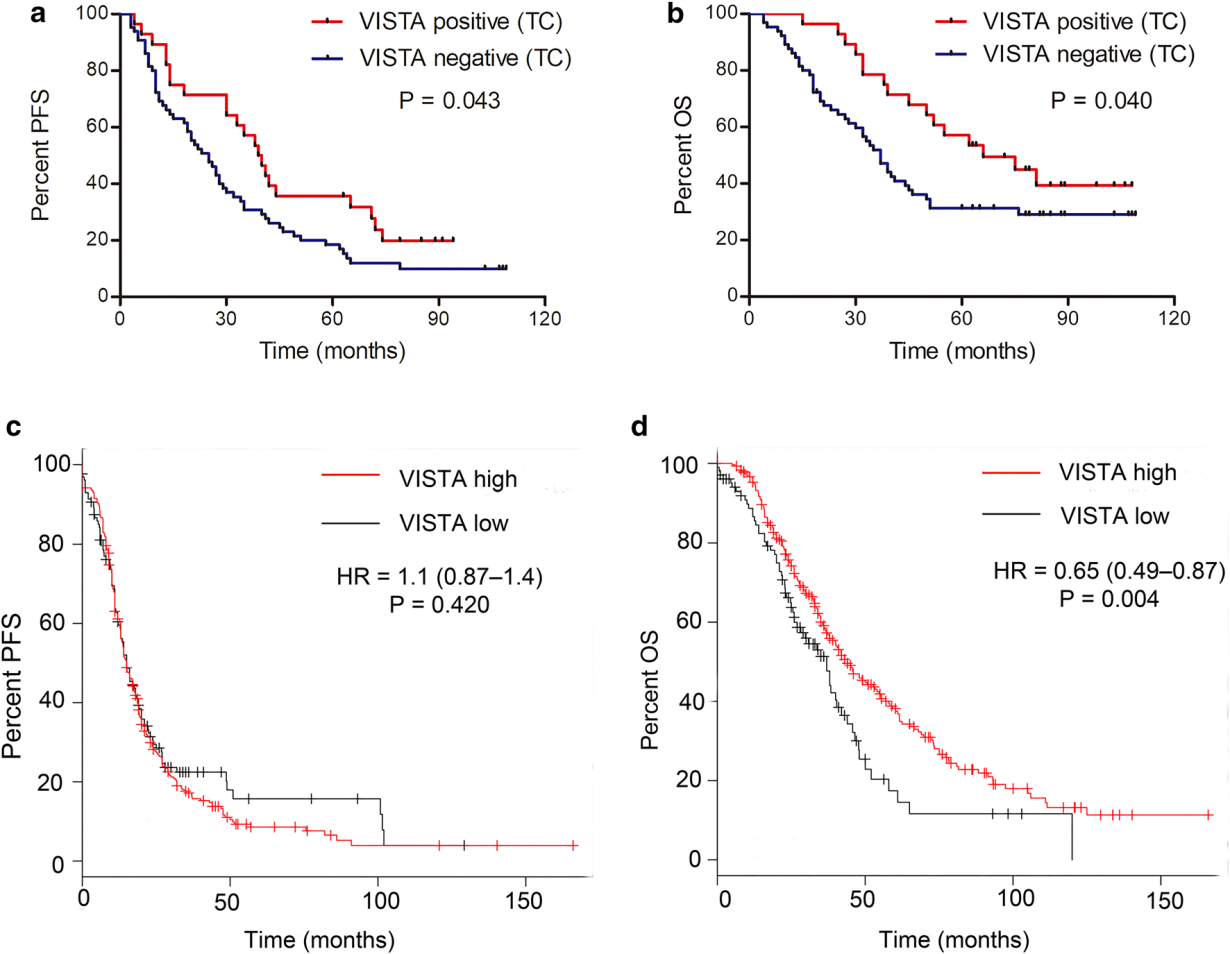
Association between V-domain Ig suppressor of T cell activation (VISTA) expression and survival in patients with high-grade serous ovarian carcinomas (HGSOC). VISTA-positive staining in tumor cells (TCs) was significantly associated with prolonged PFS (**a**) and OS (**b**). Using the Kaplan–Meier plotter database, patients with HGSOC (grades 2–3) with stage II–IV disease were subjected to analysis of OS (*n* = 405) and PFS (n = 402). High expression of VISTA mRNA (encoded by *C10orf54*) was not associated with PFS (**c**), but was significantly associated with prolonged OS (**d**). HR, hazard ratio

Survival analyses showed that VISTA-positive staining in TCs was significantly associated with prolonged PFS (*P* = 0.043, Fig. 2a) and OS (*P* = 0.040, Fig. 2b); however, VISTA expression in ICs or in all cells combined was not associated with survival (Supplementary Fig. S1). Moreover, no association between PD-L1 expression and survival was observed in these patients (Supplementary Fig. S2). We also performed survival analyses to determine the prognostic value of VISTA-encoding gene *C10orf54* expression in patients with HGSOC. The Kaplan–Meier plots revealed that high *C10orf54* mRNA expression did not influence PFS (*P* = 0.42, Fig. 2c), but was significantly associated with longer OS (*P* = 0.004, Fig. 2d). These results indicate that VISTA is associated with a favorable prognosis in patients with HGSOC.

### Biological processes modulated by genes co-expressed with C10orf54

**Fig. 3:**
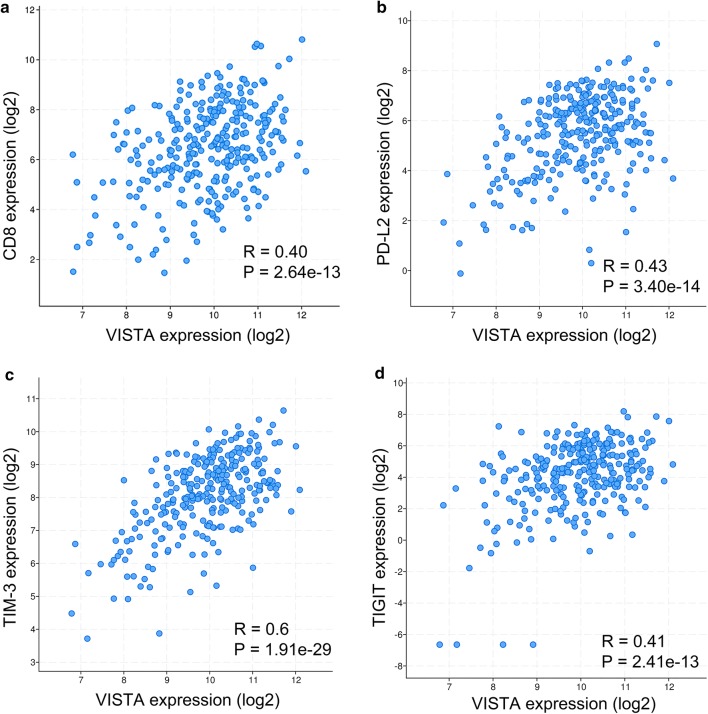
V-domain Ig suppressor of T cell activation (VISTA)-encoding gene is correlated with expression of genes that encode CD8 (**a**), programmed death ligand 2 (PD-L2) (**b**), T cell immunoglobulin and mucin domain-3 (TIM-3) (**c**) and T cell immunoreceptor with Ig and ITIM domains (TIGIT) (**d**) in ovarian serous cystadenocarcinoma samples from The Cancer Genome Atlas database

**Fig. 4:**
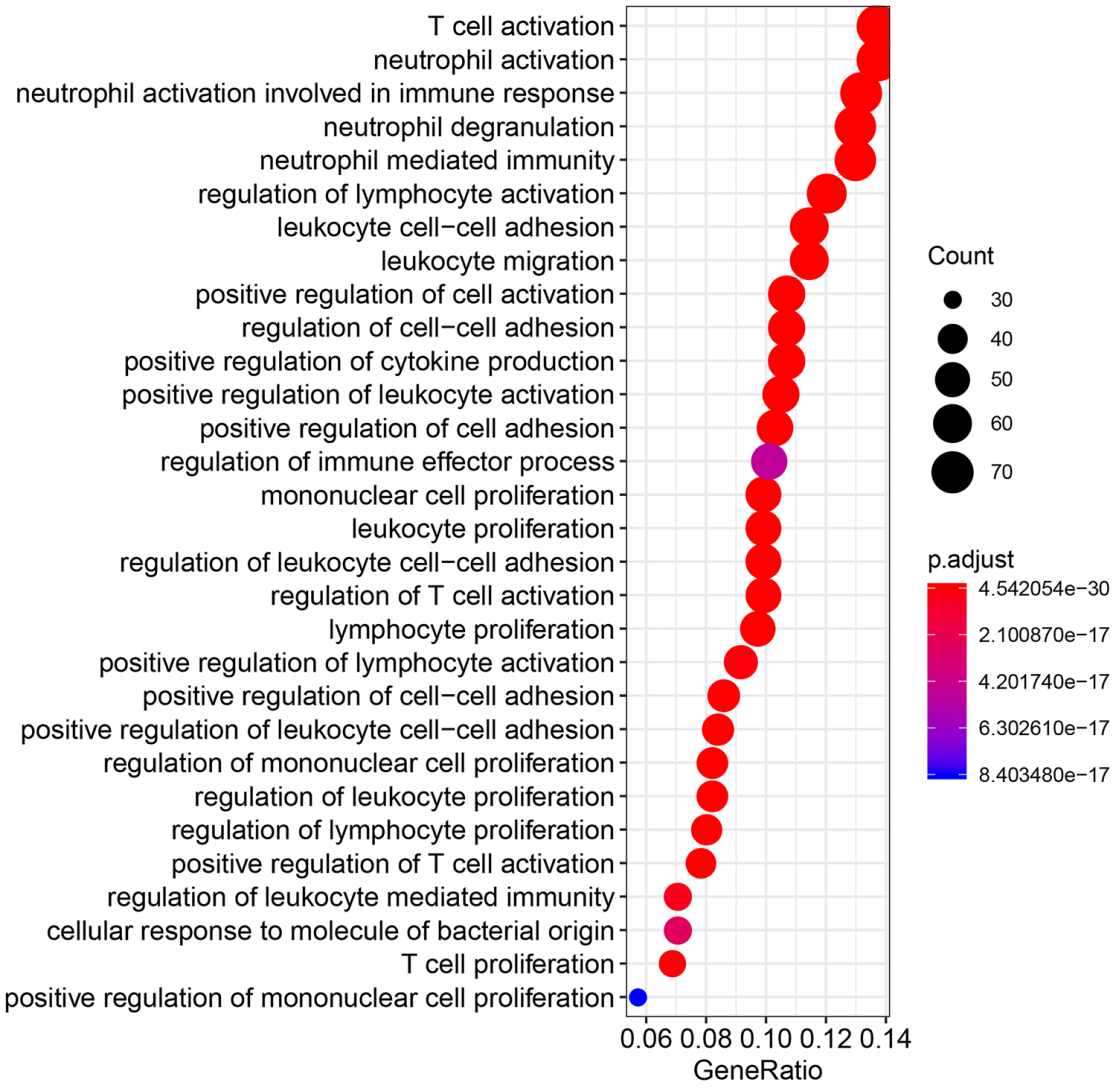
Gene-ontology biological process terms associated with V-domain Ig suppressor of T cell activation (VISTA) co-expressed genes that have Pearson correlation coefficients > 0.4

To assess the biological relevance of VISTA expression in serous ovarian cancer, we evaluated the association between the *C10orf54* gene (encoding VISTA), *CD8A* (encoding CD8), and other inhibitory immune checkpoints including *PDCD1* (encoding PD-1), *CD274* (encoding PD-L1), *PDCD1LG2* (encoding PD-L2), *HAVCR2* (encoding TIM-3), *LAG3* (encoding LAG3), and *TIGIT* (encoding TIGIT) based on TCGA mRNA expression profiles. Expression of the gene encoding VISTA was positively correlated with the expression of genes encoding CD8 (*R* = 0.40, Fig. 3a), PD-L2 (*R* = 0.43, Fig. 3b), TIM-3 (*R* = 0.6, Fig. 3c), and TIGIT (*R* = 0.41, Fig. 3d). Although VISTA was positively associated with CTLA4 (*R* = 0.38, P < 0.01), PD-1 (*R* = 0.38, *P* < 0.01), PD-L1 (*R* = 0.34, *P* < 0.01), and LAG3 (*R* = 0.32, *P* < 0.01), these correlations were weak (*R* < 0.4). Using a Pearson correlation coefficient of > 0.4 as a cutoff, we analyzed 588 genes co-expressed with VISTA for gene-ontology enrichment annotation. As shown in Fig. 4, these genes were mainly involved in modulating T cell activation, neutrophil activation, neutrophil-mediated immunity, and lymphocyte activation.

## Discussion

VISTA has attracted wide interest as a novel immune checkpoint and a potential target of cancer immunotherapy. However, little is known about its expression in ovarian cancer, especially in PD-L1-negative tumors. In the present study, we profiled VISTA and PD-L1 expression in epithelial ovarian cancer and found that VISTA protein was expressed not only in tumor-infiltrating ICs but also in TCs themselves as well as endothelial cells. We discovered that VISTA is frequently expressed in PD-L1-negative ovarian cancer and that high VISTA expression is associated with a favorable prognosis in patients with HGSOC. Moreover, our study investigated the expression of VISTA and PD-L1 in ovarian cancer using a relatively large sample size.

Using real-time polymerase chain reaction, Lines et al. found that VISTA was predominantly expressed in human placental and hematopoietic tissues, as well as in tissues that exhibit markedly high leukocyte infiltration [28]. We also found that normal human organs other than lymphoid tissues and placental trophoblastic cells did not express VISTA at the protein level [13]. Although VISTA protein has been detected in gastric cancer, oral squamous cell carcinoma, NSCLC, colorectal carcinoma, hepatocellular carcinoma, endometrial cancer, ovarian cancer, melanoma, and gestational trophoblastic neoplasia, its expression and relationship with patient survival vary according to the type of cancer [13–21]. While VISTA was mainly expressed by tumor-infiltrating lymphocytes, it was only present in 8.8% of gastric cancer cells and in 19.4–22.8% of NSCLC lesions [15, 16]. We previously reported that VISTA protein was widely overexpressed in 98.2% of all the gestational trophoblastic neoplasia [13]. Mulati et al. reported that VISTA was expressed in 84 (91.3%) of the 92 ovarian cancer tissues they investigated, and that there was no difference in survival as a function of VISTA expression [20]. Contrary to their results, we found that 75 (51.4%) of the 146 ovarian cancer tissues we investigated expressed VISTA protein in TCs and/or tumor-infiltrating ICs. Furthermore, we found that VISTA expression in TCs (but not in ICs) was significantly associated with prolonged PFS and OS in patients with HGSOC. The discrepancy between these studies may be attributed to the use of different VISTA antibodies and variable sample sizes with distinct stages and pathological distributions. Recent studies demonstrated that VISTA expression in TCs (but not ICs) was associated with significantly longer OS in patients with hepatocellular carcinoma and NSCLC [16, 21], which correspond to our findings in patients with HGSOC. Contrary to these results, Kuklinski et al. reported that VISTA expression in tumor-infiltrating inflammatory cells was correlated with worse disease-specific survival in patients with primary cutaneous melanoma [19]. These data suggest that VISTA expressed in TCs and ICs may exert different functions. However, the exact role of VISTA expressed on TCs remains unknown, and there is an urgent need to further clarify the significance and possible function of VISTA in the tumor immune environment.

Although the immune checkpoint molecules PD-L1 and VISTA belong to the B7 family, they play nonredundant roles in suppressing T cell activation and immune regulation [8]. In the present study, we found that VISTA was expressed in 46.6% of PD-L1-negative ovarian cancers, indicating that this protein might serve as an alternative immune checkpoint that suppresses T cell activation. VISTA expression was associated with that of PD-L1, and corresponded to a favorable prognosis in patients with HGSOC. However, no association between PD-L1 expression and survival was observed in our study. The prognostic significance of PD-L1 in ovarian cancer remains controversial. Hamanishi et al. reported that PD-L1 was associated with poor prognosis in patients with this disease [29], whereas Webb et al. found that PD-L1 expression was correlated with a favorable prognosis in patients with HGSOC [30]. Moreover, no association was observed between PD-L1 expression and the survival of patients with HGSOC in Mills et al.’s study [31]. Consistent with our results, a recent meta-analysis showed that PD-L1 protein expression was not associated with the survival of patients with ovarian cancer. In contrast, VISTA appears be an indicator of favorable prognosis in patients with HGSOC, although this needs to be verified in larger cohorts.

In our study, we found a significant positive correlation between *C10orf54* and genes such as *TIM*-*3, TIGIT*, and *PDCD1LG2,* which are responsible for tumor immune escape and immune suppression in human cancers. Similarly, Xie et al. found a significantly positive correlation between the expression of *C10orf54* and that of *TIGIT, HAVCR2, BTLA, CD274*, and *PDCD1* in colorectal carcinoma [17]. Furthermore, Villarroel-Espindola et al. found a consistent and prominent association between VISTA, PD-1, and PD-L1 in NSCLC at both the protein and mRNA levels [16]. Taken together, these data suggest that VISTA might contribute to immune escape in some other tumor types and could represent an effective target for immunotherapy in patients with other cancers. However, our findings suggest that this may not be the case for patients with ovarian cancer. Abiko et al. found that interferon-γ secreted by CD8-positive lymphocytes upregulated PD-L1 on ovarian cancer cells and promoted tumor growth. However, the mechanisms through which VISTA expression is regulated in the ovarian cancer microenvironment remain unknown. Therefore, additional studies are warranted to further elucidate the molecular regulation and mechanism of VISTA in ovarian cancer.

Despite our novel findings, our study had some limitations including those inherent to a retrospective study. Given the heterogeneity of tumor markers, TMA sections may not be representative of the entire tumor insofar as PD-L1 and VISTA expression. Moreover, we had a relatively small number of non-serous ovarian cancer samples; therefore, we did not analyze the relationship between VISTA and survival in patients with this tumor subtype. Furthermore, it was difficult to assess the expression of VISTA in the different subgroups of tumor-infiltrating ICs and to analyze the co-expression of VISTA and PD-L1 using immunohistochemistry. Future studies using multiplexed quantitative immunofluorescence are warranted to elucidate the expression and function of VISTA within the tumor immune microenvironment.

In conclusion, ours is the first investigation of VISTA and PD-L1 expression in a single large-size cohort of patients with ovarian cancer. Our study showed that VISTA was frequently expressed in PD-L1-negative ovarian cancer specimens and that VISTA expression in TCs, but not in ICs, was associated with the expression of genes that modulate tumor immune escape on one hand, but was also associated with prolonged survival in patients with HGSOC on the other. Further studies are urgently needed to explore the regulation and functions of VISTA in ovarian cancer to identify patients eligible for intensive treatments based on their expected prognoses.

## Electronic supplementary material

Below is the link to the electronic supplementary material.

Supplementary material 1 (PDF 204 kb)

## Abbreviations

CTLA4: Cytotoxic T-lymphocyte-associated protein 4
FIGO: International federation of gynecology and obstetrics
HGSOC: High-grade serous ovarian carcinoma
IC: Immune cell
LAG3: Lymphocyte activation gene-3
TCGA: The cancer genome atlas
TC: Tumor cell
TIGIT: T cell immunoreceptor with Ig and ITIM domains
TIM-3: T cell immunoglobulin and mucin domain-3
TMA: Tumor tissue microarray
VISTA: V-domain Ig suppressor of T cell activation
