# Supplementary material for: VISTA expression is associated with a favorable prognosis in patients with high-grade serous ovarian cancer
Source: Cancer Immunol Immunother. 2019 Nov 28;69(1):33–42. doi: 10.1007/s00262-019-02434-5 (PMC6949319; doi:10.1007/s00262-019-02434-5)

Supplementary Figures

Fig. S1 No association was observed between V-domain Ig suppressor of T cell activation (VISTA) expression in immune cells (ICs) or all cells combined (tumor cells, ICs, and endothelial cells) and survival in patients with high-grade serous ovarian carcinomas. (a) Association between VISTA expression in ICs and progression-free survival (PFS); (b) association between VISTA expression in ICs and overall survival (OS), (c) association between VISTA expression in all cells and PFS, (d) association between VISTA expression in all cells and OS.

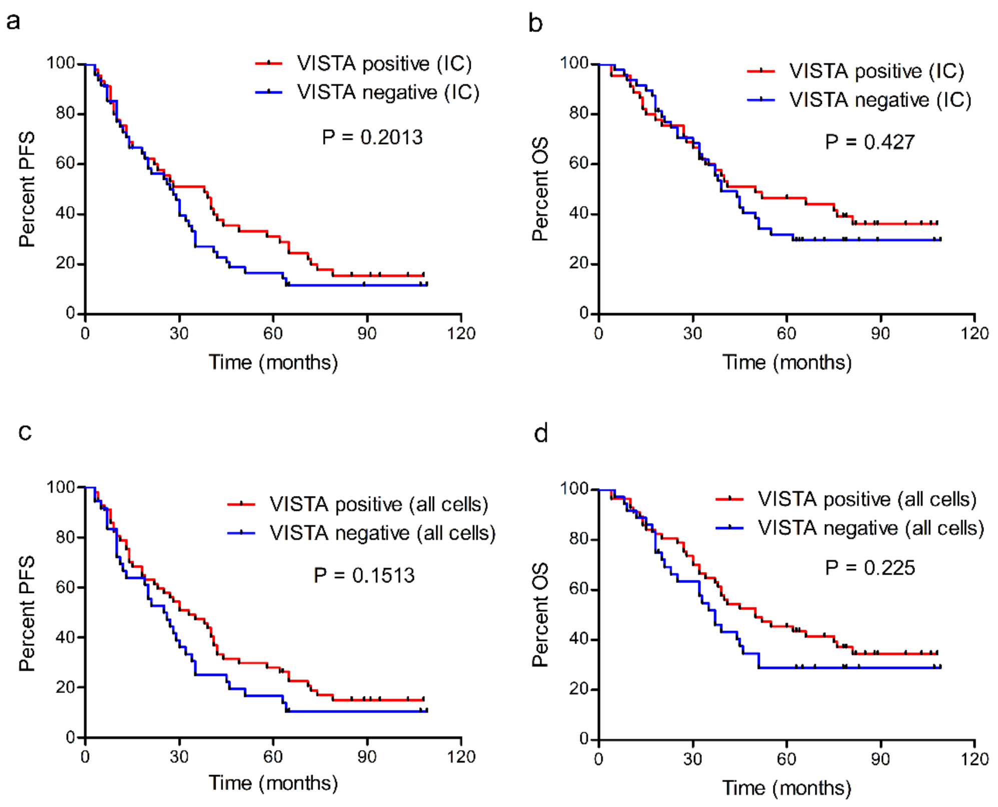

Fig. S2 No association was observed between programmed cell death-ligand 1 (PD-L1) expression and (a) progression-free survival (PFS) or (b) overall survival (OS) in patients with high-grade serous ovarian carcinomas.

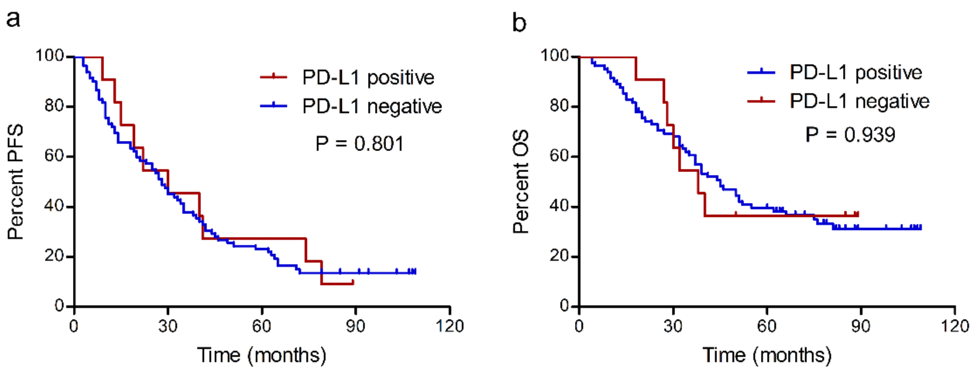

Supplement: Supplementary file 1 — Supplementary material 1 (PDF 204 kb) [file 262_2019_2434_MOESM1_ESM.pdf]
